# Chlorogenic acid protects PC12 cells against corticosterone-induced neurotoxicity related to inhibition of autophagy and apoptosis

**DOI:** 10.1186/s40360-019-0336-4

**Published:** 2019-09-09

**Authors:** Xiaowen Shi, Nian Zhou, Jieyi Cheng, Xunlong Shi, Hai Huang, Mingmei Zhou, Haiyan Zhu

**Affiliations:** 1 Center for Chinese Medical Therapy and Systems Biology, Institute for Interdisciplinary Medicine Sciences, Shanghai University of Traditional Chinese Medicine, Shanghai, 201203 China; 2 Department of Microbiological and Biochemical Pharmacy, School of Pharmacy, Fudan University, Shanghai, 201203 China; 3 Department of Cardiology, Second Affiliated Hospital of Jiaxing University, Jiaxing, 314000 Zhejiang China; 4 School of Pharmacy, Shanghai University of Traditional Chinese Medicine, Shanghai, 201203 China

**Keywords:** Chlorogenic acid, Autophagy, Neuroprotection, Corticosterone, Depression

## Abstract

**Background:**

There are evidences that chlorogenic acid (CGA) has antidepressant effects, however the underlying molecular mechanism has not been well understood. The aim of the study was to explore the neuroprotective effect of CGA on corticosterone (CORT)-induced PC 12 cells and its mechanism, especially the autophagy pathway.

**Methods:**

PC12 cells were incubated with CORT (0, 100, 200, 400 or 800 μM) for 24 h, cell viability was measured by MTT assay. PC12 cells were cultured with 400 μM of CORT in the absence or presence of CGA (25 μg/ml) for 24 h, morphologies and specific marker of autophagosome were observed by transmission electron microscope (TEM) and confocal immunofluorescence microscopy, respectively. In addition, PC12 cells were treated with different doses of CGA (0, 6.25, 12.5, 25 or 50 μg/ml) with or without CORT (400 μM) for 24 h, cell viability and changes in the morphology were observed, and further analysis of apoptotic and autophagic proteins, and expression of AKT/mTOR signaling pathway were carried out by Western blot. Specific inhibitors of autophagy 3-Methyladenine (3-MA) and chloroquine (CQ) were added to the PC12 cells cultures to explore the potential role of autophagy in CORT-induced neuronal cell apoptosis.

**Results:**

Besides decreasing PC12 cell activity, CORT could also induce autophagy and apoptosis of PC12 cells, while CGA could reverse these effects. In addition, CGA treatment regulated AKT/mTOR signaling pathway in PC12 cells. CGA, similar to 3-MA and QC, significantly inhibited CORT-induced apoptosis in PC12 cells.

**Conclusions:**

Our results provide a new molecular mechanism for the treatment of CORT-induced neurotoxicity by CGA, and suggest CGA may be a potential substance which is can alleviate depression.

## Background

Chlorogenic acid (3-O-caffeoylquinic acid) is an abundant polyphenol compound in human diet. One of the major commercial sources of CGA is the extracts of traditional Chinese medicine *Eucommia ulmoides* Oliver (*E. ulmoides*)*,* which has been demonstrated to be effective in the treatment of various central nervous system (CNS) diseases [1, 2] including neuroprotection [3], improving learning and memory [4, 5] through its various beneficial effects. Thus, as the main active compound of *E. ulmoides*, CGA has been used to treat various CNS diseases, such as depression [6, 7]. At present, although studies have shown that the chlorogenic acid-enriched extract from *E. ulmoides* exhibit potent antidepressant effects in tail suspension test of KM mice (200 and 400 mg/kg/day, orally administered for 7 days) [8], the underlying molecular mechanism of CGA’s antidepressant-like effects is unclear.

The stress response of the hypothalamic–pituitary–adrenocortical (HPA) axis with a significant rise of glucocorticoid levels has been one of the most thoroughly studied biological systems linked to the pathogenesis of depression [9–12]. CORT, the last effector of the HPA axis, is a principal glucocorticoid secreted in response to stress, and it could decrease serotonin (5-hydroxytryptamine, 5-HT) release and lead to neurodegeneration when chronic exposure to the stress level of CORT. The neurotoxicity of rat adrenal pheochromocytoma (PC12) cells can be induced by high concentrations of CORT, which has been extensively adopted as an in vitro model to investigate the impairment of neurons and depression-like syndromes [13–15].

There are increasing evidences showing that autophagy and apoptosis are involved in depression [16, 17]. Autophagy is considered to be one of the cytoprotective mechanisms by which excessive or damaged organelles are degraded, and it plays a homeostatic role at basal levels. However, excessive activation of autophagy is detrimental to normal proteins and organelles, even leading to cell death [18, 19]. Apoptosis is a type of programmed cell death that aimed to eliminate dying cells during cell proliferation or differentiation. Apoptosis plays an important role in the development and maintenance of homeostasis in multicellular organisms, it has been reported that inappropriate or excessive apoptosis is implicated in many diseases [20]. More importantly, apoptosis has a complex interplay with autophagy [21]. At the molecular level, apoptosis and autophagy share some regulatory elements, including PI3K/AKT/mTOR pathway [22], beclin1 [23], MAPK pathway [24], Bcl-2 family and p53 [25]. The external stress that leads to the activation or suppression of these regulatory elements will impact both autophagy and apoptosis. Furthermore, dysregulation of autophagic pathways, such as the mammalian target of rapamycin (mTOR) signaling pathway, has been implicated in many neurodegenerative diseases [26–28]. In addition, a large number of studies have shown that neuronal apoptosis and autophagy intervention may be an important part of the pathological process of depression. For example, reduction of hippocampal autophagy can ameliorate depression-like behavior in rats [29], and inhibition of neuronal apoptosis regulated by the AKT pathway has neuroprotective effects on chronic unpredictable mild stress (CUMS)-induced depression models [30]. Thus, the biological functions of autophagy and apoptosis in depression are worthy of investigation. Thus, the biological functions of autophagy and apoptosis in depression are worthy of investigation.

Although CGA showed antidepressant-like effect in our previous study [6], the underlying molecular mechanism has not been well understood. In this study, we investigated the neuroprotective activity and associated potential mechanisms of CGA in CORT-injured PC12 cells based on its effects on autophagy.

## Methods

### Cell culture and treatment

PC12 cells (MXC306, Shanghai Meixuan Biological Science and Technology Ltd., China) were cultured in high glucose DMEM (Corning, USA) and 10% heat-inactivated fetal bovine serum (Invitrogen, CA, USA) supplemented with 100 U/ml penicillin and 100 g/ml streptomycin (Beyotime Institute of Biotechnology, Haimen, China) at 37 °C in 5% CO_2_. For all experiments, cells in the exponential phase of growth were used. Plated PC12 cells were incubated with 100–800 μM of CORT with a purity of 92% (Sigma-Aldrich, St Louis, MO, USA) for 24 h to determine the appropriate damage concentration. CGA with a purity of 98% (Yuanye, Shanghai, China) was dissolved in dimethyl sulfoxide (DMSO). The final DMSO concentration was < 0.1% (v/v). To study the neuroprotective effect of CGA, six treatment groups were used, including control group, 400 μM of CORT, and 400 μM of CORT plus 6.25, 12.5, 25, or 50 μg/ml of CGA. Then, cells were co-incubated with CORT and CGA for 24 h.

### Reagents and antibodies

3-Methyladenine (3-MA) and chloroquine (CQ) (Sigmae-Aldrich, St. Louis, MO, USA) were prepared and diluted with DMEM. Cyto-ID Green dye was purchased from Enzo Life Sciences, Inc. (Farmingdale, NY, USA). 3- (4, 5-dimethylthiazol-2-y1) -2, 5-diphenyltetrazolium bromide (MTT) was purchased from Biotech Well (Shanghai, China). Anti-LC3B (D11), anti-phospho-mTOR (Ser2448), anti-mTOR, anti-phospho-AKT (Ser473), anti-AKT, and PARP (46D11) were purchased from Cell Signaling Technology (Danvers, MA, USA). The secondary antibodies horseradish peroxidase (HRP)-conjugated goat anti-mouse and anti-rabbit immunoglobulinG (IgG) were obtained from Biotech Well (Shanghai, China).

### Cell viability assay

Cell viability assay was performed using a MTT assay. PC12 Cells were seeded in 96-well plates and cultured, then treated with the MTT solution and incubated at 37 °C for 4 h, and then the medium was removed and 100 μl DMSO added to each well dissolved to formazan. The optical density (OD) was measured at an absorbance wavelength of 570 nm. Cell viability was expressed as a percentage of control cells. As it was reported, the MTT assay is a sensitive assay with excellent linearity up to ∼10^6^ cells per well, and subtle changes in metabolic activities can lead to large changes in MTT [31]. Therefore, the MTT assay was applied to test the activity of mitochondrial succinate dehydrogenase in living cells after 24 h of administration, to reflect the effect of the drug on cell activity.

### Confocal immunofluorescence microscopy

PC12 cells were plated in cell culture dishes with glass bottoms. After 24 h of incubation, they were divided into vehicle group, CORT (400 μM) group, CORT (400 μM) plus CGA (25 μg/ml) group, and positive group. Cells in the positive group were treated with 50 nM autophagy inducer rapamycin for 18 h using the Cyto-ID® Autophagy Detection Kit according to the manufacturer’s protocol. Briefly, cells were washed twice with 1 × assay buffer, and then treated with Cyto-ID® Green dye and Hoechst 33342 at 37 °C for 30 min. After incubation, cells were washed with 1 × assay buffer and immediately analyzed with an Olympus fluorescence microscope.

### Transmission electron microscopy

Cells were fixed with 2% glutaraldehyde in a cell culture medium for 15 min, and then fixed in 2% glutaraldehyde with 0.1 M NaCacodylate/HCL (pH 7.4) for 30 min. After that, cells were washed three times in 0.2 M NaCacodylate/HCL (pH 7.4), fixed with 1% OsO_4_–0.15 M NaCacodylate/HCL (pH 7.4) for 30 min, dehydrated in an increasing gradient of ethanol and finally polymerized at 60 °C for 48 h. Samples were cut and analyzed with a JEM 1230 transmission electron microscope (JEOL, Peabody, MA).

### Western blot

PC12 cells were harvested, washed twice with cold phosphate-buffered saline (PBS), and lysed in RIPA lysis buffer (Beyotime Institute of Biotechnology, Haimen, China) for 30 min on ice. The lysates were centrifuged, and the supernatants were collected. The protein concentration was determined by the bicinchoninic acid (BCA) method. Equivalent amounts of protein were separated by sodium dodecyl sulfate-polyacrylamindegelelectrophoresis (SDS-PAGE) and then transferred to poly vinylidene fluoride membranes. The membranes were blocked in Tris-buffered saline and Tween 20 (TBST) containing 3% bovine serum albumin (BSA) for 1 h at room temperature, and subjected to primary antibodies at 4 °C overnight and then the second antibody conjugated with horseradish peroxidase for 2 h at room temperature. The membranes were washed in TBST and the signals were visualized using an enhanced chemiluminescence detection kit (Pierce, Rockford, IL). Densitometric values of protein bands were quantified by the IQuant TL software (GE Healthcare, USA). The target gene and housekeeping genes in the displayed western blot map reflected the same membrane source data, without washing and re-hybridizing. The experiment was repeated 3 times independently (*n* = 3), and grayscale data analysis is an average analysis of three replicates.

### Statistical analysis

GraphPad Prism 5 was used for all statistical analyses. The results were expressed as mean ± SD. Statistical comparisons were performed using One-way ANOVA, and *P* values < 0.05 were considered statistically significant.

## Results

### CORT induced autophagy and neurotoxicity in PC12 cells

**Fig. 1:**
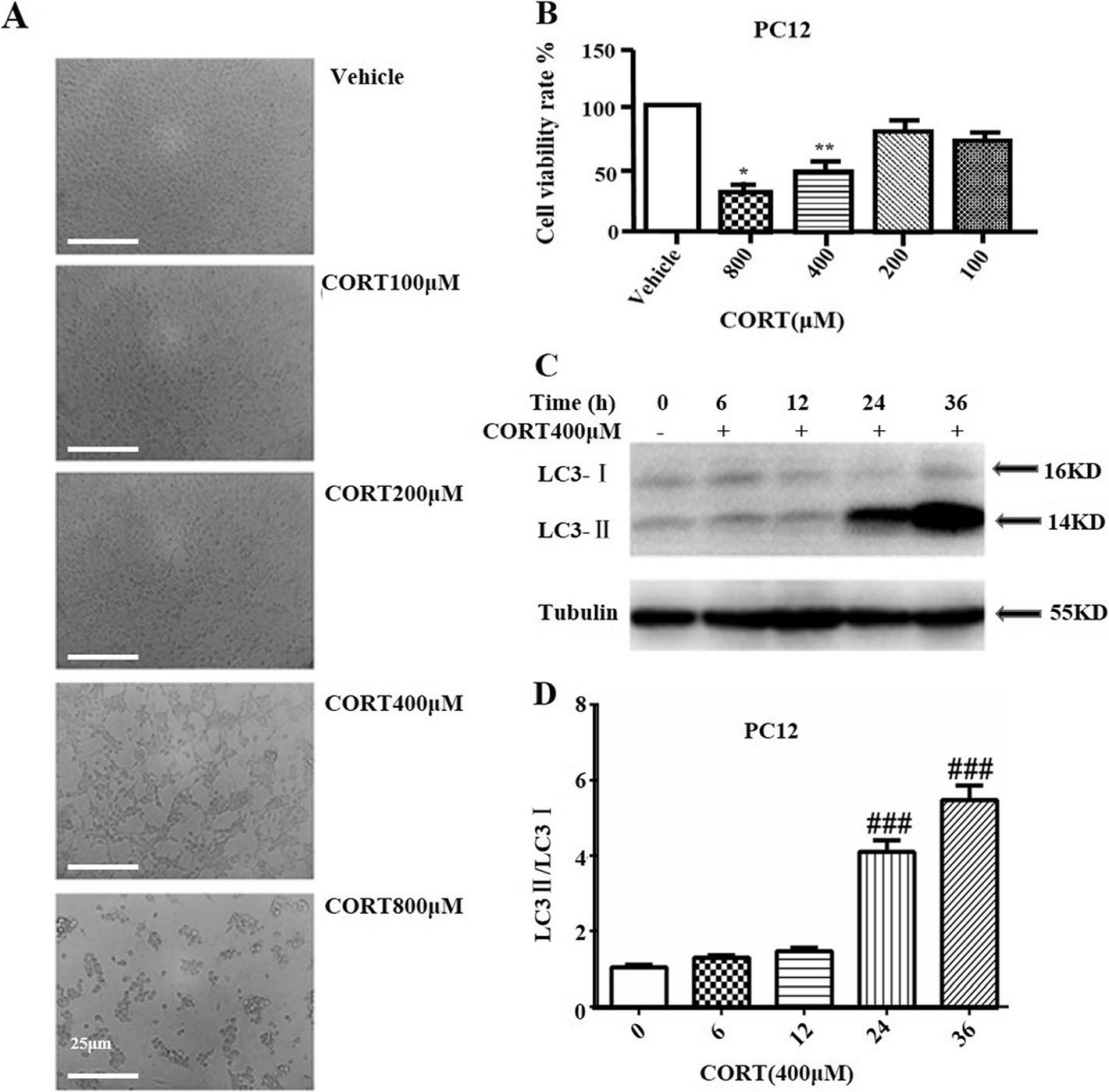
Autophagy and neurotoxicity were triggered by CORT in PC12 cells. **a** Changes in the morphology and number of PC12 cells were observed by optical microscope (Scale bar, 25 μm). **b** PC12 cell viability was measured by MTT assay (*n* = 3). **c** PC12 cells lysates were subjected to Western blotting using LC3 and tubulin antibodies (*n* = 3). **d** Densitometric values of LC3-II and LC3-I were quantified using the AlphaEaseFC software. Data are presented as mean ± SD. ^#^ *P* < 0.05, ^##^ *P* < 0.01, ^###^ *P* < 0.001 compared with the vehicle group. * *P* < 0.05, ** *P* < 0.01, *** *P* < 0.001 compared with the CORT group

To investigate the induction effect of CORT on autophagy and neurotoxicity of PC12 cells, cells were treated with different concentrations of CORT for 24 h, respectively, and cell viability was determined by MTT assay. As shown in Fig. 1a、1B, the morphology and viability of PC12 cells vary with the concentration of CORT. PC12 cells after treatment were irregular in shape, poor in adhesion, and even shedding. Treatment with 400 μM of CORT for 24 h resulted in a decrease of cell viability to approximately 50%, which was selected for subsequent experiments. The fact of 400 μM CORT induces cytotoxicity in PC12 cells is available in similar experiments [15, 32]. Moreover, the results of Western blot analysis suggest that the LC3-II / LC3-I ratio increases along with the time, and is related to the degree of cell damage (Fig. 1c, d). These results clearly demonstrated that CORT has a significant effect on autophagy and neurotoxicity of PC12 cells.

### CGA protected PC12 cells from CORT-induced neurotoxicity

**Fig. 2:**
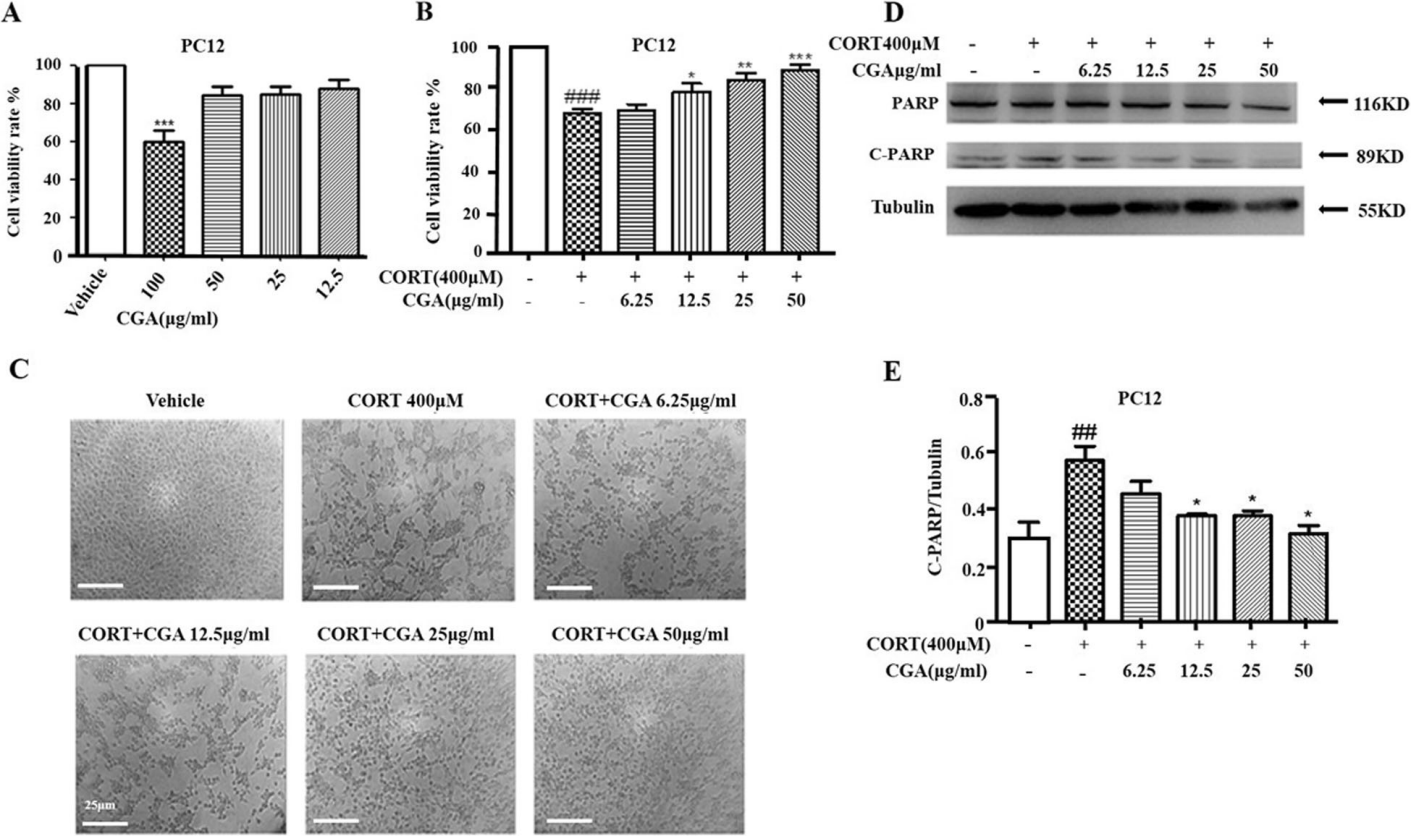
CGA protected against CORT-induced neurotoxicity in PC12 cells. **a** PC12 cells were cultured with various concentrations of CGA for 24 h, and cell viability was measured by MTT assay (*n* = 3). **b** Cell viability was determined by MTT assay (*n* = 3). **c** Changes in the morphology of PC12 cells were observed by optical microscope (Scale bar, 25 μm). **d** Changes in the expression of C-PARP were examined by Western blot. **e** Densitometric values of C-PARP were quantified using the AlphaEaseFC software. Data are presented as mean ± SD. ^#^ *P* < 0.05, ^##^ *P* < 0.01, ^###^ *P* < 0.001 compared with the vehicle group. * *P* < 0.05, ** *P* < 0.01, *** *P* < 0.001 compared with the CORT group

It is necessary to examine the toxicity of CGA on PC12 cells prior to further assays. PC12 cells were treated with various doses of CGA (12.5–100 μg/ml) for 24 h. No significant changes were observed in the viability of cells treated with 12.5–50 μg/ml of CGA, while treatment with 100 μg/ml of CGA resulted in an obvious effect on cell viability (Fig. 2a) Thus, 100 μg/ml of CGA was excluded in subsequent studies. Treatment with CORT alone resulted in a decrease in cell viability compared with the control group, whereas addition of CGA significantly decreased CORT-induced toxicity in a concentration-dependent manner (Fig. 2b, c). These results demonstrated that CGA inhibited CORT-induced neurotoxicity in PC12 cells.

In order to examine the protective effect of CGA on PC12 cells, apoptosis protein levels were examined by western blot analysis. Figure 2d and e showed that C-PARP level significantly decreased in the co-treatment groups of CORT and different doses of CGA compared with CORT treated cells. These results indicated that CORT markedly induced apoptosis in PC12 cells, whereas CGA significantly decreased cell apoptosis.

### CGA suppressed CORT-induced autophagy in PC12 cells

**Fig. 3:**
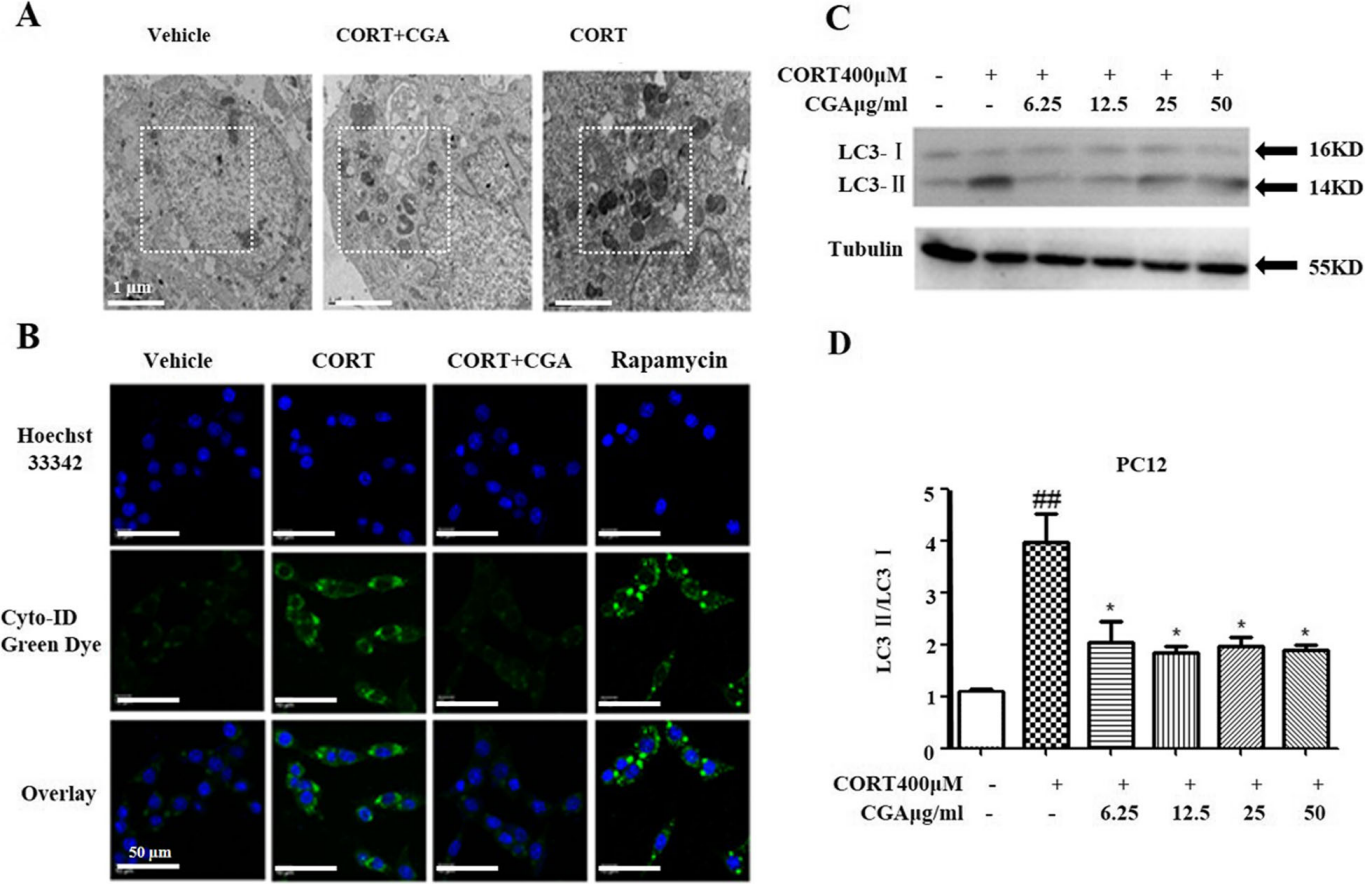
CGA suppressed CORT-induced autophagy in PC12 cells. **a** PC12 cells morphologies were observed by TEM, (Scale bar, 1 μm). **b** PC12 cells stained with Cyto-ID Green autophagy dye and analyzed by confocal microscopy. Untreated cells served as the vehicle control and cells treated with 50 nM rapamycin for 12 h served as the positive control, (Scale bar, 50 μm). **c** Changes in the expression of LC3 were examined by Western blot in PC12 cells (n = 3). **d** Densitometric values of LC3-II and LC3-I were quantified using the AlphaEaseFC software. Data are presented as mean ± SD. ^#^ *P* < 0.05, ^##^ *P* < 0.01, ^###^ *P* < 0.001 compared with the vehicle group. * *P* < 0.05, ** *P* < 0.01, *** *P* < 0.001 compared with the CORT group

We speculated that CGA could inhibit the formation of autophagosome and thus contributed to the survival of CORT treated PC12 cells. To address this hypothesis, we first examined the morphology of PC12 cells by TEM. CORT treated cells showed numerous vacuoles and electron-dense inclusions (Fig. 3a). However, treatment of CGA inhibited the formation of autophagosome in CORT treated PC12 cells.

Autophagy was detected using an autophagy detection kit by fluorescence microscopy. Cyto-ID green fluorescence dye was a specific marker of autophagic vacuoles [33]. Figure 3b showed strong green fluorescence in the cytoplasm of CORT treated PC12 cells, but less fluorescence in PC12 cells treated with both CORT and CGA, and much less fluorescence in vehicle cells.

In addition, we also examined the expression of LC3-I and LC3-II by Western blot. Autophagosome marker LC3-II reflects autophagy activity, and detection of LC3 has become a reliable method for monitoring autophagy and autophagocyte-related processes [34]. The results revealed that LC3-II was up-regulated in CORT treated PC12 cells, while CGA inhibited the transformation of LC3-I to LC3-II (Fig. 3c). Figure 3d showed that the LC3-II/LC3-I ratios were significantly decreased in PC12 cells treated with both CORT and CGA compared with PC12 cells treated with CORT alone.

### CORT/CGA treatment regulated the AKT/mTOR signaling pathway in PC12 cells

**Fig. 4:**
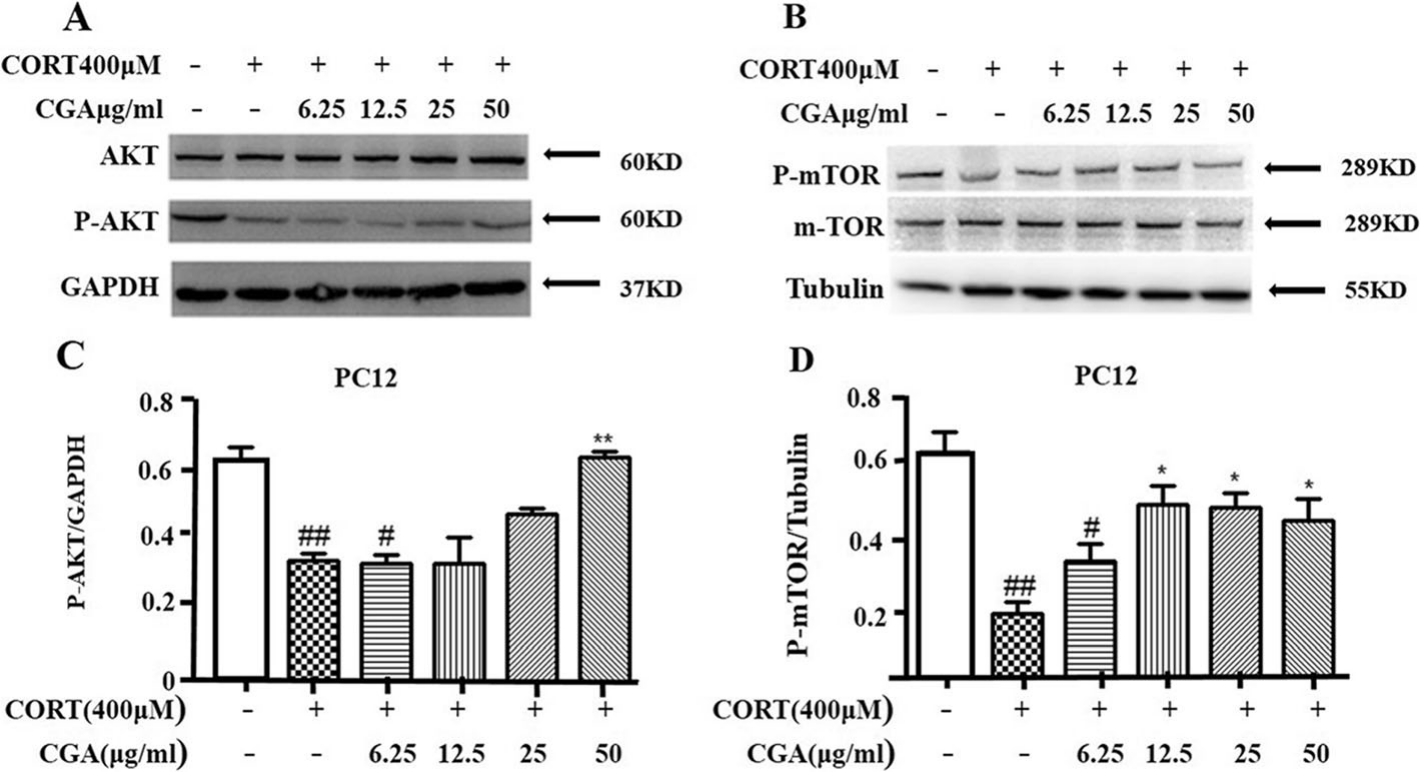
AKT/mTOR pathway was involved in the suppression of CORT-induced autophagy of PC12 cells by CGA. **a** Changes in the expression of P-AKT were examined by Western blot in PC12 cells (n = 3). **b** Changes in the expression of P-mTOR were examined by Western blot in PC12 cells (n = 3). **c** Densitometric values of P-AKT were quantified using the AlphaEaseFC software. **d** Densitometric values of P-mTOR were quantified using the AlphaEaseFC software. Data are presented as mean ± SD. ^#^ *P* < 0.05, ^##^ *P* < 0.01, ^###^ *P* < 0.001 compared with the vehicle group.* *P* < 0.05, ** *P* < 0.01, *** *P* < 0.001 compared with the CORT group

We have demonstrated that CGA is involved in protecting PC12 cells from CORT-induced damage. Next, in order to study the potential molecular mechanism of CGA inhibiting CORT-triggered autophagy, we examined the levels of P-AKT and P-mTOR in PC12 cells to determine whether AKT/mTOR signaling pathway is involved in CORT-induced autophagy. Western blotting demonstrated that the P-AKT and P-mTOR levels were significantly reduced in PC12 cells following CORT treatment, but significantly increased in PC12 cells treated with CGA (Fig. 4a, b, c, and d), thus all the results together indicated that CORT-induced autophagy could be mediated by the inhibition of AKT and subsequent inhibition of mTOR expression in PC12 cells.

### Inhibition of autophagy by CGA protected against CORT-induced apoptosis

**Fig. 5:**
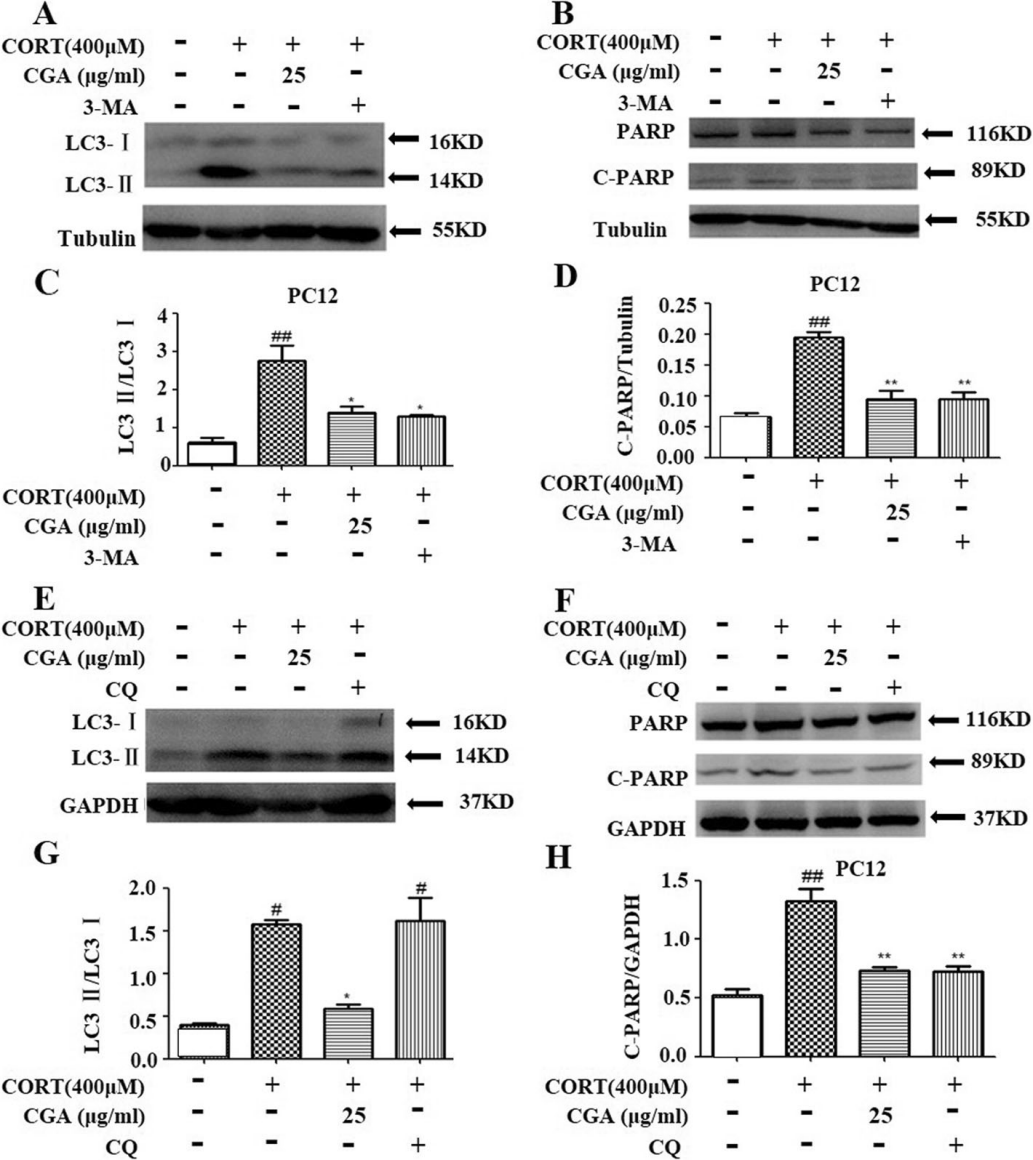
The inhibition of autophagy by CGA protected against CORT-induced apoptosis. **a** PC12 cells were incubated with or without CORT in the presence of CGA or autophagy inhibitor 3-MA for 24 h. Changes in the expression of LC3 were examined by Western blot (n = 3). **b** PC12 cells were incubated with or without CORT in the presence of CGA or autophagy inhibitor 3-MA for 24 h. Changes in the expression of C-PARP were examined by Western blot (n = 3). **c** Densitometric values of LC3-II and LC3-I were quantified using the AlphaEaseFC software. **d** Densitometric values of C-PARP were quantified using the AlphaEaseFC software. **e** PC12 cells were incubated with or without CORT in the presence of CGA or autophagy inhibitor CQ for 24 h. Changes in the expression of LC3 were examined by Western blot (n = 3). **f** PC12 cells were incubated with or without CORT in the presence of CGA or autophagy inhibitor CQ for 24 h. Changes in the expression of C-PARP were examined by Western blot (n = 3). **g** Densitometric values of LC3-II and LC3-I were quantified using the AlphaEaseFC software. **h** Densitometric values of C-PARP were quantified using the AlphaEaseFC software. Data are presented as mean ± SD. ^#^ *P* < 0.05, ^##^ *P* < 0.01, ^###^ *P* < 0.001 compared with the vehicle group.* *P* < 0.05, ** *P* < 0.01, *** *P* < 0.001 compared with the CORT group

To assess the role of autophagy in CORT-induced neuronal cell death, two autophagy inhibitors, 3-MA and CQ, were used to inhibit CORT-induced autophagy. 3-MA is an inhibitor of phosphatidylinositol 3-phosphate kinase (PI3K) which could reduce LC3-II protein level, and CQ could inhibit the fusion between autophagosomes and lysosomes and elevate LC3-II protein level [35, 36]. In the pretest, we tested 4 concentrations, and the results showed that 25 μg/ml CGA could exert neuroprotective effects on CORT-induced nerve injury, such as significantly increasing cell viability compared with the CORT group, significantly reducing the amount of C-PARP and the conversion rate of LC3I to LC3II. However, the in vitro activity test of traditional Chinese medicine often has HOOK effect. Therefore, we chose a medium dose of 25 μg/ml with strong activity to compare the effects of autophagy inhibitors 3MA and CQ on autophagy and apoptosis. Western blot analyses clearly showed that pre-treatment with 3-MA resulted in a decrease in the expression of LC3-II and cleaved PARP in CORT-treated cells. Similarly, these two proteins reduced significantly in the CGA group (Fig. 5a, b, c, and d); while pre-processing with CQ resulted in an increase in LC3-II protein level and a decrease in cleaved PARP in CORT-treated cells. In contrast, both LC3-II and cleaved PARP levels were reduced in the CGA-administered group. (Fig. 5e, f, g, and h). With the treatment of autophagy inhibitors 3-MA and CQ, the apoptosis of PC12 cells induced by CORT was decreased, and CGA produced similar effects. Thus, these results suggest that inhibition of CORT-induced apoptosis by CGA may be related to inhibition of autophagy in PC12 cells.

## Discussion

In recent years, the protective effects of CGA on cells have been confirmed [37], and the protective effect of CGA on hepatocyte injury is related to autophagy [38]. In this study, the results showed that CGA had protective effects on CORT-induced neurotoxicity in PC12 cells. More specifically, CGA enhanced cell viability and inhibited CORT-induced autophagy and apoptosis in PC12 cells. It also regulated the activity of AKT-mTOR signaling pathway. Thus, the neuroprotective effects of CGA may be associated with the reduction of neuronal cell apoptosis and autophagy.

Previous studies have suggested that a high concentration of CORT can induce cellular damage in PC12 cells, which simulate the pathomechanism of depression in vitro [14], and this can be reversed by antidepressants [39]. Consistent with these findings, 400 μM CORT treatment for 24 h, the cell viability decreased to approximately 50% compared with the control in our study, confirming the CORT’s neurotoxicity. By contrast, CGA significantly increased the cell viability. These findings suggested that CGA may have the potential to resist depression.

Apoptosis is closely related to neurotoxicity. Studies have shown that inhibition of apoptosis can attenuate neurotoxicity in PC12 cells [40, 41]. In the early stages of apoptosis, C-PARP is cleaved by Caspase-3 from PARP into two fragments, which is considered to be a characteristic feature of apoptosis [42]. In our study, CORT increased C-PARP level in PC12 cells (Fig. 2d). Both Fig. 5b and Fig. 2d showed a tendency for CGA to inhibit apoptosis, as well as significantly inhibition of the expression of C-PARP elevated by CORT, and there was no significant inhibitory activity against uncleaved RARP. Thus, inhibiting the apoptosis of PC12 cells may be an effective method to prevent the progression of neurotoxicity.

Autophagy is a protein degradation pathway in which intracellular membrane structures package part of the cytoplasm to form a double-membrane vesicle (autophagic vesicles or autophagosomes) that fuses with lysosomes forming the autophagolysosomes (APs) [43, 44]. In this study, treatment of 400 μM CORT for 24 h in PC12 cells induced typical autophagy due to cellular damage. We investigated the autophagic vacuoles presenting in PC12 cells and detected the abundance of fluorescence in treated cells. Western blot analysis also showed a conversion of LC3 I to LC3 II in response to CORT exposure. All of these results demonstrated that CGA efficiently inhibited autophagy in PC12 cells exposed to CORT.

Moreover, both autophagy and apoptosis are meditated by the AKT/mTOR signaling pathway. [45]. It is well known that this pathway is important for widely divergent cellular processes including cell proliferation, cell death, differentiation and metabolism [46]. Studies have shown that inhibition of this pathway may lead to increased autophagy under pathophysiological stress [47]. More importantly, AKT/mTOR signaling pathway, as a negative regulatory of autophagy, is involved in antidepressants counteract CORT-induced depression-like behavior [48–50]. Our results show that CORT treatment decreases AKT/mTOR pathway activity, but this effect is reversed by CGA. Thus, the increase of autophagy after CORT treatment is closely linked to the inhibition of AKT/mTOR pathway, demonstrating the pathway plays a pivotal role in autophagy.

Autophagy and apoptosis, two different types of programmed cell death, are not mutually exclusive processes and show synergy and resistance in many models [51]. To explore the relationship between autophagy and apoptosis, two autophagy inhibitors were used in this study to block CORT-induced autophagy, 3-MA (autophagosome formation inhibitor) and CQ (autophagy recycling inhibitor). The results demonstrate that the inhibition of autophagy and apoptosis showed a consistent trend. Importantly, depression is associated with neuronal apoptosis, and neuroprotection is an important strategy for antidepressants [52]. Thus, these results suggest that regulation of autophagy and apoptosis is a promising strategy for interfering with the physiological and pathological processes of depression and provide insights into drug discovery. However, we initially evaluated the effects of drugs on cell viability through the MTT method, and did not further and systematically study the synergistic effects of the two drugs. We regret to admit that this is a shortcoming in our research work and needs to be improved in the future work.

## Conclusions

In summary, the protective effect of CGA on CORT-induced neurotoxicity in PC12 cells is related to inhibition of autophagy and apoptosis, and involves the AKT/mTOR signaling pathway. The results of the current study may contribute to improve the understanding of the neuroprotective effects of various compounds in *E. ulmoides*. However, further experimental studies in vivo are required to clarify the mechanism of CGA on CORT-induced neurotoxicity and neurological disorders.

## Data Availability

All data generated or analyzed during this study are included in this published article [and its supplementary information files].

## Abbreviations

3-MA: 3-Methyladenine
AKT: Protein kinase B; h: hour
BCA: Bicinchoninic acid
BSA: Bovine serum albumin
CGA: Chlorogenic acid
CORT: Corticosterone
CQ: Chloroquine
DMSO: Dimethyl sulfoxide
HRP: Horseradish peroxidase
IgG: Immunoglobuling
mTOR: Mammalian target of rapamycin
MTT: 3- (4, 5-dimethylthiazol-2-y1) -2, 5-diphenyltetrazolium bromide
OD: Optical density
PBS: Phosphate-buffered saline
TBST: Tris-buffered saline and Tween 20
TEM: Transmission electron microscope
